# The Nicotinic Receptor Alpha7 Impacts the Mouse Lung Response to LPS through Multiple Mechanisms

**DOI:** 10.1371/journal.pone.0121128

**Published:** 2015-03-24

**Authors:** Elena Y. Enioutina, Elizabeth J. Myers, Petr Tvrdik, John R. Hoidal, Scott W. Rogers, Lorise C. Gahring

**Affiliations:** 1 Geriatric Research, Education and Clinical Center (GRECC), Veterans Affairs Medical Center, Salt Lake City, Utah, United States of America; 2 Department of Internal Medicine, Division of Geriatrics, University of Utah School of Medicine, Salt Lake City, Utah, United States of America; 3 Department of Pathology, Division of Microbiology and Immunology, University of Utah School of Medicine, Salt Lake City, Utah, United States of America; 4 Department of Human Genetics, University of Utah School of Medicine, Salt Lake City, Utah, United States of America; 5 Department of Internal Medicine, Division of Pulmonary Medicine, University of Utah School of Medicine, Salt Lake City, Utah, United States of America; 6 Veterans Affairs Medical Center, Salt Lake City, Utah, United States of America; 7 Department of Neurobiology and Anatomy, University of Utah School of Medicine, Salt Lake City, Utah, United States of America; School of Medicine and Health Sciences, University of North Dakota, UNITED STATES

## Abstract

The nicotinic acetylcholine receptor alpha7 (α7) is expressed by neuronal and non-neuronal cells throughout the body. We examined the mechanisms of the lung inflammatory response to intranasal (i.n.) lipopolysaccharide (LPS) regulated by α7. This was done in mice using homologous recombination to introduce a point mutation in the α7 receptor that replaces the glutamate residue 260 that lines the pore with alanine (α7^E260A^), which has been implicated in controlling the exceptional calcium ion conductance of this receptor. The α7^E260A^ mice exhibit normal inflammatory cell recruitment to the blood in response to i.n. LPS administration. This differs from the α7knock-out (α7^KO^) in which upstream signaling to initiate the recruitment to the blood following i.n. LPS is significantly impaired. While hematopoietic cells are recruited to the bloodstream in the α7^E260A^ mouse, they fail to be recruited efficiently into both the interstitium and alveolar spaces of the lung. Bone marrow reconstitution experiments demonstrate that the responsiveness of both CD45^+^ and CD45^-^ cells of the α7^E260A^ mouse are impaired. The expression of several pro-inflammatory cytokine and chemokine RNAs including TNFα, IL-1α, Ccl2 and Cxcl10 are decreased in the α7^E260A^ mouse. However, there is a substantial increase in IL-13 expression by CD45^-^ lung interstitial cells in the α7^E260A^ mouse. Our results support the conclusion that α7 functional pleiotropy contributes to modulating the tissue response to an inflammatory insult through impacting upon a variety of mechanisms reflecting the individual cell composition of the lung.

## Introduction

Among the most abundant agents in cigarette smoke (CS) is nicotine, which in addition to being the addictive substance of CS also modulates inflammatory responses [1–4]. In the body nicotine interacts with ionotropic nicotinic acetylcholine receptors (nAChR) that are expressed by both neuronal and non-neuronal cells that together modulate a multitude of cellular responses which in many instances govern how normal and pathological processes progress [5]. Physiological interaction with nicotine from CS, or other sources such as electronic-cigarettes (e-cigarettes), occurs first through the oral cavity followed by the lung. A prominent nAChR subtype expressed in the lung whose function is implicated in the response to nicotine is alpha7 (α7) [6–9]. This nicotinic receptor is well-recognized to modulate both central and peripheral neurotransmission, but it also modifies inflammation through mechanisms that directly alter non-neuronal cell responses including immune cells, epithelial cells and adipocytes [10]. This impact is explained in part by the unique ion channel properties of α7 that in addition to the normal sodium/potassium current include an exceptional permeability to calcium [5]. In this case, the calcium current is sufficient to modulate multiple calcium-mediated intracellular processes which have been suggested to resemble metabotropic-like properties in addition to its contribution to depolarization responses [11]. Thus, the broad distribution of α7 expression must be considered when defining how activation of this receptor impacts on responses throughout the body.

The role of α7 in inflammation has been supported by studies examining the α7^KO^ mouse that elicits greater inflammatory cytokine responses in a tissue specific manner including more cellular infiltration into a site of inflammation [3,12–14]. Notably, α7-modulatory processes can be imparted through: 1) through parasympathetic nerve function; 2) direct impact on hematopoietic cells such as macrophages that express this receptor; and 3) cells and tissues such as keratinocytes of the skin where parasympathetic innervation is absent [12,13,15]. However, as would be predicted, should these processes converge in a single tissue, the impact of α7 on the tissue response through modulation of different pathways may not always be obvious in the α7^KO^. Further, the possibility of functional compensation of α7 in the knock-out animal by other nAChRs or other cellular signaling systems could obscure the specific contribution of this receptor makes to the inflammatory process. In the lung previous studies identified α7 expression by multiple resident non-hematopoietic cells such as lung macrophages derived from the hematopoietic system and bronchial epithelium and endothelium [6,8,16–18]. Lung macrophages fall into two categories broadly defined as alveolar macrophages (AM), which are resident in bronchial alveolar lavage fluid (BALF) of normal mice, and interstitial tissue macrophages (IM) of the lung tissue parenchyma [19]. AMs are distinguished by their expression of the CD11c marker which is traditionally associated with dendritic cells [20–22] in contrast to IMs that express predominantly the CD11b (MAC1) surface marker and are infrequently CD11c^+^. Lung airway inflammation is characterized by an influx of macrophages as well as granulocytes such as polymorphonuclear cells (PMN) into both the alveolar space and interstitial tissue, which is an integral part of the acute response to an airway irritant [23]. The initiation of lung inflammation also involves the cellular activation of epithelial cells which also express α7 receptors [6,8,24]. Maouche et al. [24] found that α7 expressed by epithelium plays a role in the regulation of the cystic fibrosis transmembrane conductance regulator (CTFR) which in the lung regulates mucus transport. In the case of CS exposure, the effect of nicotine on these α7 expressing epithelial cells may contribute to the pathogenesis of chronic lung disease [24]. A secondary effect of CS/nicotine of great concern is through maternal smoking which is a leading cause of sudden infant death syndrome (SIDS) and the α7 receptor has been suggested to be strongly expressed in lung epithelial cells of such individuals [25]. Further, in the α7^KO^ mouse, basal cell hyperplasia has been reported [26]. Thus within different lung compartments several different cell subtypes express α7 making them potential targets of nicotine’s impact on the system response.

To begin the dissection of how α7 contributes to the individual organ-specific inflammatory response, we have used a genetic approach to develop new tools that provide us with the specificity and sensitivity for measuring α7 expression by lung cells in the mouse. Through homologous recombination we have generated mice in which a bi-cistronic internal ribosome entry sequence (IRES):tau-GFP expression cassette (α7^G^) or an IRES:Cre recombinase expression cassette (α7^Cre^) has been added to the 3’ end of the α7-transcript [27,28]. These constructs dramatically improve the ability to detect α7 gene expression and offer the important benefit of doing so while retaining normal genomic context, copy number, and minimal perturbations to normal gene expression and function. The application of this model has already revealed novel insights into many possible roles α7 may have both during development and in the adult [27–30]. In the present study we have extended this model to define the role of a point mutation reported to be associated with ablated α7-calcium channel permeability [31–33] to the inflammatory response in the lung. The α7 channel normally conducts both sodium/potassium and calcium, each with a specific function contributing to neurotransmission (Na^+^/K^+^) and cell signaling pathways (calcium). The uniquely high permeability of α7 to calcium has been reported to rely on the presence of a glutamic acid that is present at position 260 in the mouse α7 protein [5,31–35]. The introduction of a dominant E260A point mutation into α7^G^ (α7^E260A:G^) using the precision of homologous recombination removes the ‘charge’ lining the intracellular pore of α7 to essentially abolish the calcium current without impact on other receptor functions [31–33]. The ability to distinguish the impact of this point mutation in α7 processes can be accomplished through directly comparing the α7^E260A:G^ response to the genetically-matched wild-type α7^G^ control mice. We have applied these new mouse models to examine the expression of α7 in the lung, and to define the contribution of α7 to the response by resident and infiltrating cells to an intranasal inflammatory challenge.

Our findings provide evidence that α7 impacts upon the inflammatory process in the lung through simultaneously affecting different mechanisms regulated by both hematopoietic cells and local non-hematopoietic cells. First, the α7^KO^ mouse responds significantly less well to an inflammatory challenge to the lung, which is unlike the enhanced inflammatory response observed in the skin of these mice challenged with croton oil or ultraviolet radiation [12,13]. A significantly reduced lung inflammatory response to i.n. LPS was also observed in the α7^E260A:G^ mouse. Both the α7^KO^ and the α7^E260A:G^ mice demonstrate poor influx of inflammatory cells into the BALF and a greatly reduced recruitment of cells in to the interstitial lung tissue of LPS (intranasal, i.n.) challenged mice. Second, reconstitution of irradiated WT recipient mice with either α7^G^ or α7^E260A:G^ bone marrow shows that the donor α7^G^ cells respond to i.n. LPS with an influx of inflammatory cells into the lung but α7^E260A:G^ bone marrow reconstituted mice are poorly-responsive to this inflammatory stimulus. We demonstrate that this alteration in responsiveness may be due to altered chemokine and cytokine production by both CD45^+^ as well as the CD45^-^ cells of the lung. Third, while hematopoietic cell expression of key inflammatory cytokines and chemokines is modified by in the α7^E260A:G^ mice, there is a robust increase in the expression of the cytokine IL-13 by the non-hematopoietic compartment of the lung. We discuss the role of α7 expression on cells of the lung, both CD45^+^ and CD45^-^ cells, and how the control by α7 signaling of an emerging cytokine profile contributes to inflammatory processes that are initiated in the lung and are likely to be modified by those who are at risk to pathologies through the varied processes resulting from nicotine exposure.

## Materials and Methods

### Antibodies

Anti-Ly6G (clone IA8, BD Bioscience Pharmingen, San Jose, CA), Ly6C (clone AL-21, BD Bioscience Pharmingen), CD11b (clone M1/70, BD Bioscience Pharmingen), Gr1 (RB6–8C5, BD Bioscience Pharmingen), FcR (Fc block, BD Bioscience Pharmingen), F480 (clone BM8, eBioscience, San Diego, CA), CD11c (clone HL3, BD Bioscience Pharmingen), CD45 (clone 30-F11, BD Bioscience Pharmingen) were used per manufacturers protocols.

### Animals

The mice were housed and used for this study in accordance with protocols approved in advance by the Institutional Animal Care and Use Committee at the University of Utah (Protocol Number #12–06001). In all cases animals were maintained according to the Guide for the Care and use of Laboratory Animals of the National Institutes of Health. Each experiment used groups of 3–5 mice that were age, gender and strain matched. The Rosa26-loxP-enhanced yellow fluorescent protein reporter (*ROSA26*-YFP, Jackson laboratory Stock Number 006148) and α7^Cre:YFP^ mouse lines were previously described [27]. All α7^Cre:YFP^ mice were routinely monitored for the ratio of YFP^+/-^ cells in the blood by Flow Cytometry (FACS). Mice containing approximately 15–25% of YFP^+^ cells in the blood (unless otherwise stated) were used in experiments.

### Generation of E260A Chrna7-IRES-tauGFP mice

The *Chrna7*-E260A allele (α7^E260A^) harbors missense mutations that replace the glutamate residue 260 with alanine, that selectively and severely restricts the permeability of α7 receptors to calcium in open state [5,31–34]. These mutations were introduced in mouse embryonic stem cells by gene targeting. The targeting vector is identical to the plasmid described previously [27], except for two nucleotide changes (CT>GG) in exon VII inducing E260A codon conversion. Briefly, the targeting vector was constructed by subcloning a 9440-bp genomic DNA fragment, harboring the last four exons of the *Chrna7* gene, from a 129S6/SvEvTac BAC clone pulled out of the RPCI-22 BAC Library. The nucleotide changes required for E260A conversion were introduced by PCR with the following primers: forward 5’-GACTCTGGAgccAAAATCTCTCTTGGTAAGTGTCCATGT-3’ and reverse 5’-AGAGATTTTggcTCCAGAGTCTGCAGGCAGCAAGAATACCA-3’ (mutagenic nucleotides are indicated in lowercase). Next, we inserted a hemagglutinin (HA) epitope tag (YPYDVPDY) in the C-terminus of the *Chrna7* gene and the IRES-tauGFP-FRT-neo-FRT reporter and selection cassette in the 3’ untranslated region. The targeting vector was completely sequenced and linearized with XhoI. Electroporation into 129 ES cells and genetic screening by the loss-of-allele assay and long range PCR was performed by the Mouse Biology Program (MBP) at the University of California in Davis (Project A7E260A-KI). Three ES clones (out of 96) harbored the desired genetic modifications and passed karyotyping tests. One of the male chimeras generated with these ES cells transmitted the tauGFP reporter as well as the targeted mutations, as confirmed by sequencing of PCR-amplified exon VII (primers LR-Chrna7_SDM-R 5’-GAACCAGGGGTTTATGATGTTGTGAG-3’ and LR-Chrna7_SDM-F3 5’-GACTAAGACGTTGTATCCCTGAAGAAAGG-3’; sequencing primer LR-Chrna7_SDM-S 5’-GCCCATAGTGTCCTGTAGAGCTAGC-3’). This founder was mated with FLPe deleter mice to remove the neomycin selection marker and was subsequently bred to homozygosity as before. PCR genotyping is performed with a cocktail of four PCR primers (5’- CCAACACATG ATGAGCACC-3’ [WT fw], 5’- TGCCGAGTAC AATGATATGC C-3’ [WT rev], 5’- GCCTTCTATC GCCTTCTTGA C-3’ [neo], 5’- ACAACCACTA CCTGAGCACC-3’ [GFP]), yielding a 483-bp product with WT DNA, a 395-bp product with the allele in which neo has been deleted, and a 286-bp product when neo is still present. Automated genotyping service for this allele is available from Transnetyx (using probes “Chrna7–2 WT”, “eGFP” and “Chrna7–7 Mut”).

### Bone marrow reconstitution

Cells were isolated from the femurs and tibias of donor mice. Red blood cells were lysed and the remaining cells passed through sterile 70 μm nylon cell strainer and washed several times. During cell isolation the recipient mice (B6.SJL, CD45.1) were exposed to a split dose (2 x 6 Gy) of radiation at a 3-hour interval using a Shepherd Mark I 137Cs source (JL Shepherd and Associates, Glendale, CA, http://www.jlshepherd.com) at a dose rate of 0.8 Gy/minute. Bone marrow cells were transplanted by the retro-orbital route under tribromoethanol (Avertin) anesthesia (1:40 dilution of 1 mg/ml sterile solution, 0.3ml/mouse, i.p.) at a dose 5,000 cells/mouse in 200 μl of Saline. After cell injection, the recipient mice were placed on acidified antibiotic containing water. Analysis of reconstitution was done on a biweekly basis starting from week 2 after transplant. Blood samples were analyzed for Gr1^+^, CD11b^+^, B220^+^ and CD8^+^ cells expressing donor markers.

### Intranasal (i.n.) LPS administration to mice

Prior to the procedure, all mice were anesthetized by intraperitoneal (i.p.) injection of 0.2–0.3ml of 1:40 dilution of tribromoethanol (Avertin, 1mg/ml, Sigma, St. Louis, MO) in sterile saline. After being completely sedated, experimental mice (3–5 per group) received an i.n. inoculum of *E*. *coli* LPS 055:B5 (250 μg/mouse in 30 μl of saline, 15 μl/nostril, Sigma, St. Louis, MO). Control mice received an inoculum of 30μl saline (15 μl/nostril). In pilot experiments it was determined that a positive lung response to i.n. LPS challenge correlated with 2–3 fold increase in the percentage of Gr1^+^CD11b^+^ cells in the blood of challenged mice measured 24 hrs after challenge (not shown). Mice receiving an i.n. saline inoculum did not demonstrate an increase in Gr1^+^CD11b^+^ cells in the blood at any time point tested (24 hrs to 8 days post-challenge) when compared to naïve animals. We chose intranasal administration of LPS over intratracheal administration because of the less invasive nature of this challenge that itself can be a source of inflammation especially to the trachea. Also, while many LPS studies use tissue harvested at 48 hrs, we observed that the peak of the response to i.n. administration of LPS occurred at 72 hrs (not shown).

### Bronchial alveolar lavage fluid (BALF) cell collection

Experimental and control mice were sacrificed by injection of tribromoethanol at different times after i.n. LPS challenge. BALF was collected by gently flushing lungs (8–10 times) with 1 ml of lung buffer (1x DPBS, 2% BSA, 0.05% EDTA) via insertion of a butterfly needle into the trachea. Trachea were excluded from harvested tissue.

### Interstitial cell isolation

Following BALF lavage no attempt was made to perfuse the remaining tissue. As such, the interstitial tissue cells represent both circulating cells and interstitial cells. These cells were obtained by dicing the isolated lung tissue and incubating with dissociation medium (10 ml DMEM, 500 μl DNase 1 (1 mg/ml) containing 100 μl Liberase (2.5 mg/ml)) in a water bath at 37°C for 40 min. Halfway through this incubation, tissues were gently passed through an 18-gauge needle. After incubation, cells were removed from the water bath and 20 ml of ice-cold lung buffer were added. Cells were centrifuged at 4°C for 10 min., followed by RBC lysis and washes with buffer.

### Phenotypic characterization of cells present in BALF and interstitium

One hundred to two hundred thousand nucleated cells from BALF or cells from interstitial tissues were re-suspended in 200μl of staining buffer (1x DPBS, 2% BSA, 0.1% sodium azide, 0.05% EDTA). One microgram of anti-mouse CD16/32 (FcR block) was added to each sample to block non-specific binding of monoclonal antibodies to murine FcRγ. After 15min. of incubation at 4°C, monoclonal antibodies directed against mouse CD45, CD11b, CD11c, Gr1, Ly6C, Ly6G, F4/80 (0.2–0.5 μg/sample) conjugated to the appropriate fluorophores were added to the cell samples and incubated at 4°C for 15–30 minutes. After incubation cells were washed twice with staining buffer and filtered through 70 μm nylon filters. A minimum of ten to twenty thousand events were collected using the Accuri Cell Cytometry System (Ann Arbor, MI). Data were analyzed with FCS Express software (De NOVO software, Los Angeles, CA). Gating was based on isotype controls and where indicated, on expression of the CD45 marker. Expression and gating of YFP^+^ cells was assessed with lung cells isolated from *ROSA26*-YFP mice which serve as negative controls for YFP expression. Lung interstitial and BALF cells have significant autofluorescence that we were able to significantly reduce through use of 90% attenuation filters for the Accuri flow cytometer. The attenuation filters were FL1 (CD144), FL2 (CP148), FL3 (CP164), and FL4 (CP165). Further, non-specific antibodies were used to verify the specificity of our staining.

### Interstitial CD45^+^ and CD45^-^ cell enrichment

Isolated interstitial cells were pelleted by centrifugation. The supernatants were aspirated and the cell pellets were resuspended in 90 μL of running buffer (0.2% FBS, 2mM EDTA in 1X PBS) per 10^7^ cells. CD45 mouse microbeads (Miltenyi Biotec, San Diego, CA) were added at a concentration of 10 μL microbeads per 10^7^ cells. Cells were mixed by brief vortexing and incubated for 15 minutes at 4°C. Cells were washed by adding 5 ml running buffer and centrifuged. The supernatants were aspirated and the cell pellets were resuspended in 2 mL of running buffer. Cell suspensions were passed through a 70 μm nylon mesh filters prior to separation. Interstitial CD45^+^ and CD45^-^ cells were isolated using an autoMACS cell sorter (Miltenyi Biotec). Isolated CD45^+^ and CD45^-^ fractions were routinely tested for purity. Both fractions demonstrated 92–95% purity and 3–5% 7AAD positive.

### Gene array

SABioscience mouse chemokine & receptors PCR array (cat. no. PAMM-022 and PAMM-011; QIAGEN, Inc, Valencia, CA) were used to compare the relative levels of cDNA between interstitial CD45^-^ cells or CD45+ cells from interstitium and BALF which were isolated from saline or i.n. LPS challenged (20 hrs) α7^G^ or α7^E260A:G^ mice mRNA was extracted using Qiazol (QIAGEN, Inc, Valencia, CA). cDNA was prepared using the RT2 real-time SYBR Green/Rox PCR master mix and RT2 Profiler PCR array (QIAGEN Inc.). All samples were amplified using Applied Biosystems 7900HT 384 well sequence detecting system (Life Technologies, Grand Island, NY). Results of PCR array were analyzed using the web-based RT2Profiler PCR Array Data Analysis software (version 3.5, QIAGEN).

### Cytokine/chemokine gene expression analysis

Total RNA was isolated from cells as described previously. The concentration and purity of the RNA was determined with a ND-1000 Spectrophotometer (NanoDrop Technologies, Wilmington, DE). Each RNA sample (1 μg) was DNase treated and converted to cDNA via reverse transcription PCR (Promega, Madison WI) and used to amplify cDNA (high capacity cDNA Archive Kit, Applied BioSystems, Foster City CA). 20 ng of cDNA was loaded into each well and the following TaqMan Gene Expression Assays were used: α7, IL1α, IL1β, IL-6, TNFα, IL-13, BMP6, Ccl12 and Cxcl10. Data were calculated as gene copies per 10,000 or 100,000 copies of β-actin [36].

### Statistical analysis

Statistical significance of differences observed between the experimental and control groups, which demonstrated the normal response to the inflammagen, were determined by Student’s t-test. Differences were considered as statistically significant when probability values were less than 0.05.

## Results

### Lung cell lineages exhibiting α7 expression

**Fig 1:**
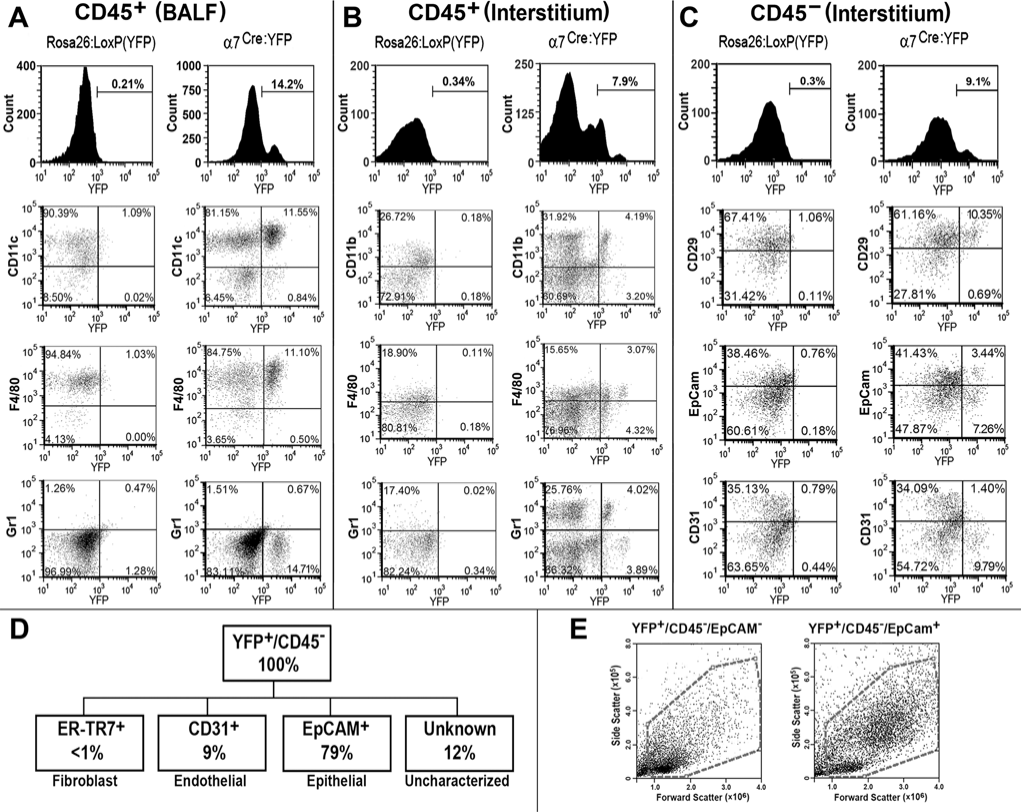
Mouse α7 lineage-marked lung cells are both CD45^+^ and CD45^-^. BALF cells were collected from groups of 3–5 mice (3–4 months old) naïve ROSA:YFP or α7^Cre:YFP^ mice (3–4 mo old). BALF cells were pooled from each genotype and then blocked with anti-CD16/CD32 antibodies (FcRγ) to prevent non-specific binding. Cells were then stained with monoclonal antibodies directed against mouse CD45, CD11c, F480 (F4/80) or Gr1 and analyzed by FACS for these markers as well as YFP expression. (A) A representative experiment shows the percent of total CD45^+^ cells in the gates as indicated. (B, C) Following removal of BALF, the remaining lung tissue (Interstitium) was digested (see Methods) and the single cell suspensions blocked and stained for to group cells into the hematopoietic (CD45^+^; B) or non-hematopoietic (CD45^-^; C) lineages. Additional markers appropriate to the respective lineage were then used including those specific to CD45^-^ interstitial cells (CD29, EpCam, and CD31) and again quantitated in terms of α7 lineage (YFP^+^ or YFP^-^). (D) The results of multiple measurements are summarized in in terms of the average percentage of α7 lineage (YFP^+^ or YFP^-^) CD45^+^ or the CD45^-^ populations including markers of fibroblasts (ER-TR7), endothelial cells (CD45-, CD31+, EpCAM^-^) and epithelial cells (CD45^-^, EpCAM^+^, CD31^-^) cells. The majority of the CD45^-^YFP^+^ interstitial cells are EpCAM^+^ that identifies epithelial cells. Some cells (12%) were not identified by the markers examined. The mean of each measurement varies by approximately 5% between experiments. (E) Examination of the forward scatter (indicator of cell size) and side scatter (indicator of granularity) of the YFP^+^/CD45^-^/EpCAM^-^ (left panel) and YFP^+^/CD45^-^/EpCAM^+^ cells establishes the difference in their respective morphology. Stained samples were analyzed by FACS using FCS Express software. In all cases each experiment was repeated at least 3 times with each experimental group consisting of 3–5 mice.

The α7 lineage positive cell (α7^lin+^) are identified in mice bred from parental crosses of the α7^Cre^ x *ROSA26*-YFP by imaging constitutive YFP expression subsequent to α7^Cre^ expression [27,28]. We previously demonstrated that α7^lin+^ cells on average constitute 10 to 25% of the cells from bone marrow, blood, spleen, and other immune tissues [28]. This ratio is present at weaning and remains stable during the lifespan of the animal [28]. In the lung, α7^lin+^ cells are also present as demonstrated by flow cytometry (Fig. 1). Both CD45^+^ and CD45^-^ cell populations harbored α7^lin+^ and α7 lineage negative (α7^lin-^) cells (Fig. 1). In similar cell populations isolated from the parental *ROSA26*-YFP control mouse strain YFP^+^ cells were not observed (Fig. 1). The total number of cells isolated from the lungs of control α7^Cre^, *ROSA26*-YFP mice and the α7^Cre:YFP^ mice, respectively, were equivalent (both for BALF and interstitial cells, not shown). Therefore, representation of the percent staining in this figure also indicates that the number of cells of each phenotype (e.g., CD11c) is equivalent. The α7^lin+^ cell population in the lung, similar to hematopoietic cells, constitutes only a subportion of the total number of cells (approximately 5 to 15% of the cells; see also [28]. Typically, the percent of α7^lin+^ cells is lower in the interstitial tissue compared to the blood and BALF (Fig. 1 and data not shown). Additional phenotyping demonstrated the α7^lin+^ cells in the BALF are CD11c^+^F480^+^ (Fig. 1B). Gr1^+^ cells (including Ly6G^+^ polymorphonuclear (PMN) cells) are not normally present in the BALF of naïve mice while interstitial CD45^+^ cells are mostly CD11b^+^Gr1^+^F480^+^ (Fig. 1B). There are few CD11c^+^ cells in the interstitial cell preparation (not shown). The CD31 (PeCAM) marker is present on endothelial cells while CD29 is associated with both endothelial cells and epithelial cells and EpCAM is present on epithelial cells but not endothelial cells. Further analysis of the CD45^-^/YFP^+^ population of cells from the α7^lin+^ mouse is shown in Fig. 1D and 1E. Of the α7^lin+^ cells (YFP^+^) that are CD45^-^ (not of hematopoietic origin), we determined the percentage of endothelial cells (CD31^+^), fibroblasts (ER-TR7^+^), and epithelial cells (EpCAM^+^). Few fibroblasts are present in this CD45^-^ population while CD31^+^ cells represent 9% of the cells. Epithelial cells represent 79% of the CD45^-^/YFP^+^ population and approximately 12% of the CD45^-^/YFP^+^ population are currently uncharacterized. Measuring forward scatter (a function of cellular size), and side-scatter which is a function of cellular granularity (Fig. 1E) shows that the majority of the epithelial cells (CD45^-^/EpCAM^+^/YFP^+^) have a different morphology that the endothelial cell population (CD45^-^/CD31^+^/YFP^+^) which one would expect for these cell types.

### Intranasal LPS administration elicits cell recruitment into the BALF and interstitial tissue

**Fig 2:**
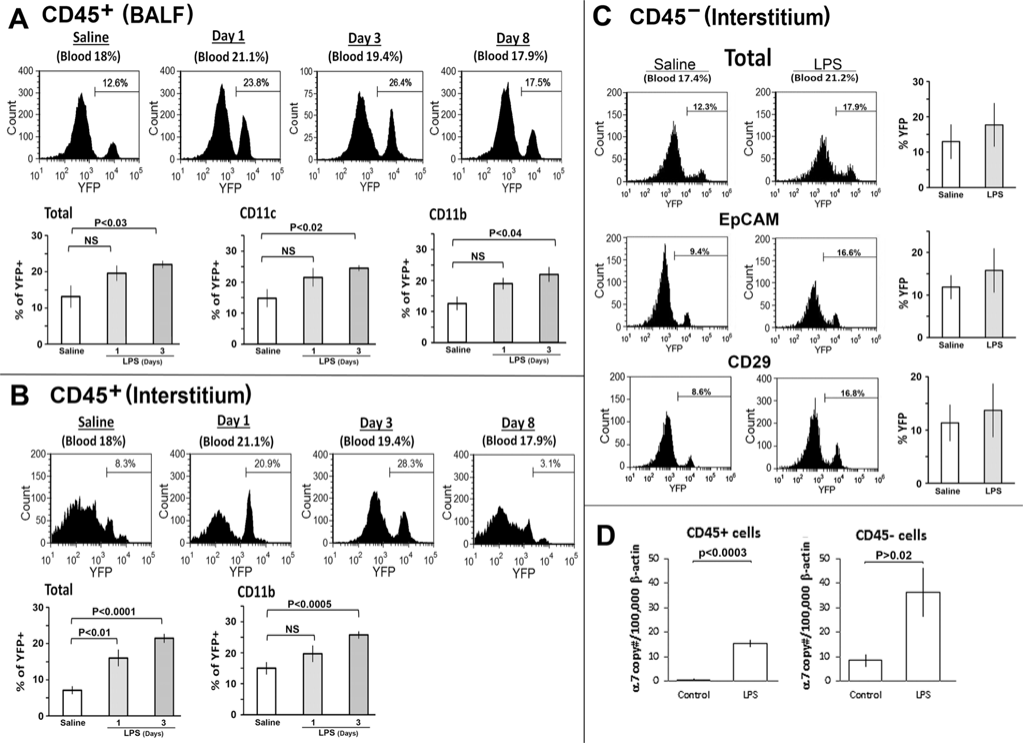
Phenotypic characteristics of BALF and interstitial cells from α7^Cre:YFP^ mice challenged with i.n. LPS. Mice expressing cells that are lineage marked for WT α7 expression (α7^Cre:YFP^, 3–5 mo, 3–5 mice per group) were challenged with i.n. LPS (250 μg/mouse in 30 μl of saline). Control mice received i.n. 30 μl of saline. With LPS administration i.n., the number of cells isolated from the BALF and interstitium (CD45^+^) increases 2 to 3 fold by 72 hrs. A minimum of twenty thousand events were collected for each sample. Histograms reflect the percent of α7^lin+^ (YFP^+^) cells that are present on the days noted. The bar graphs represent data combined from three independent experiments (3–5 mice per experimental group in these experiments) with error bars reflecting plus-minus the standard error of the mean. Significance levels were determined using the Student’s t-test and this reflects the summed results of from at least three independent experiments. (A) BALF cells were isolated, FcRγ-blocked and stained with anti-mouse CD45, CD11c, and CD11b antibodies. In these experiments BALF was collected at Days 1, 3 and 8 for comparison to the naïve (saline control) group. Quantitative results are shown for day 1 and 3, where day 3 was observed to be the optimal response to LPS. Note the selective increase in the α7^lin+^ (YFP^+^) cell percentage after LPS exposure. (B, C) Cells of the interstitium were isolated from the remaining lung tissues by enzymatic digestion and stained with anti-mouse CD45, CD11b, EpCAM, and CD29. Flow cytometric gating was on either CD45^+^ cells (B) or on CD45^-^ cells (C). The increase in percentage of CD45^-^ α7^lin+^ (YFP^+^) cell after LPS exposure in this time frame was less in comparison to the CD45^+^ population. (D) Interstitial lung CD45^+^ and CD45^-^ cells were collected 72 hrs post-challenge with either i.n. saline or LPS using CD45 microbeads and autoMacs sorting. RNA was isolated from these populations (CD45^+^ or CD45^-^). In both populations an increase in α7 RNA was observed in response to LPS administration. Combined analysis of α7 gene transcripts from three independent experiments is shown. Data are presented as mean ± SEM.

As observed in Fig. 1, naive α7^lin+^ and α7^lin-^ cells exhibit similar marker expression (also see [28]). To determine if the α7^lin+^ or α7^lin-^ differ in response to an inflammatory challenge, the response induced by intranasal administration of LPS was measured both in the blood and the different lung compartments (Fig. 2). Animal groups with similar initial α7^lin+^:α7^lin-^ cell ratios as measured in the blood were administered either saline (i.n.) or LPS (i.n.) and the lungs harvested 24 hrs to 30 days later. We found that in three independent experiments the maximal response to LPS occurs at 72 hrs (Day 3) post LPS challenge (data not shown). Studies [18] using intratracheal administration of LPS report that the peak response in terms of cellular influx occurs at 48 hours which is likely due to the more direct, but more invasive, LPS challenge. We also found that the ratio of α7^lin+^:α7^lin-^ cells present in BALF or interstitium of unstimulated animals (prior to any treatment) is less than measured in the blood. For example 18% of the cells in the blood are α7^lin+^ but in the BALF 12% are α7^lin+^ (Fig. 2A). However, the α7^lin+/lin-^ ratio begins to increase to a statistically significant value relative to the saline control in both cells of the BALF and interstitium by 72 hours after LPS stimulation (Fig. 2A; p < 0.03). By day 8 the α7^lin+^:α7^lin-^ ratio in both lung compartments returned to near pre-LPS levels where it remains to day 30 (Fig. 2A, B and data not shown). The lung samples from control (saline treated) mice were harvested at 72 hrs, and we observed no influx of inflammatory cells nor an increase in percentage of α7^lin+^ cells in the lungs at this time or at any of the saline treated points tested (Fig. 2 and not shown). YFP expression by the non-hematopoietic (CD45^-^) cells was also measured (Fig. 2C). There is a modest increase in the overall CD45^-^ α7^lin+^:α7^lin-^ ratio at 72 hours following LPS insult.

For both the CD45^+^ and CD45^-^ population of α7^lin+^ cells, it was determined whether the origin of the increase in the α7^lin+^:α7^lin-^ ratio reflects a change in α7 transcripts in response to using qPCR. In all experiments there was an increase in α7 transcripts in both CD45^+^ and CD45^-^ cell populations at the 72 hour optimal time post-LPS challenge (Fig. 2). This increase was not measurable at earlier 24 or 48 hour times (not shown). In the CD45^+^ population the increase in α7 transcripts, while significant (p<0.02), was small (~10 copies per 100,000 of beta-actin). Given this small change, the results collectively favor the difference in number of CD45 ^+^α7^lin+^ cells in the lung is due to the preferential presence of α7^lin+^ cells into the interstitium and alveolar space in response to the inflammatory insult. This conclusion is supported further by the results from the CD45^-^ population. In these cells despite the increase of 25–35 copies α7/100,000 beta-actin transcripts, only a modest doubling of the α7^lin+^ cells is observed which was not statistically significant.

### Comparing the response to LPS by α7^KO^ and α7^E260A:G^ mice

**Fig 3:**
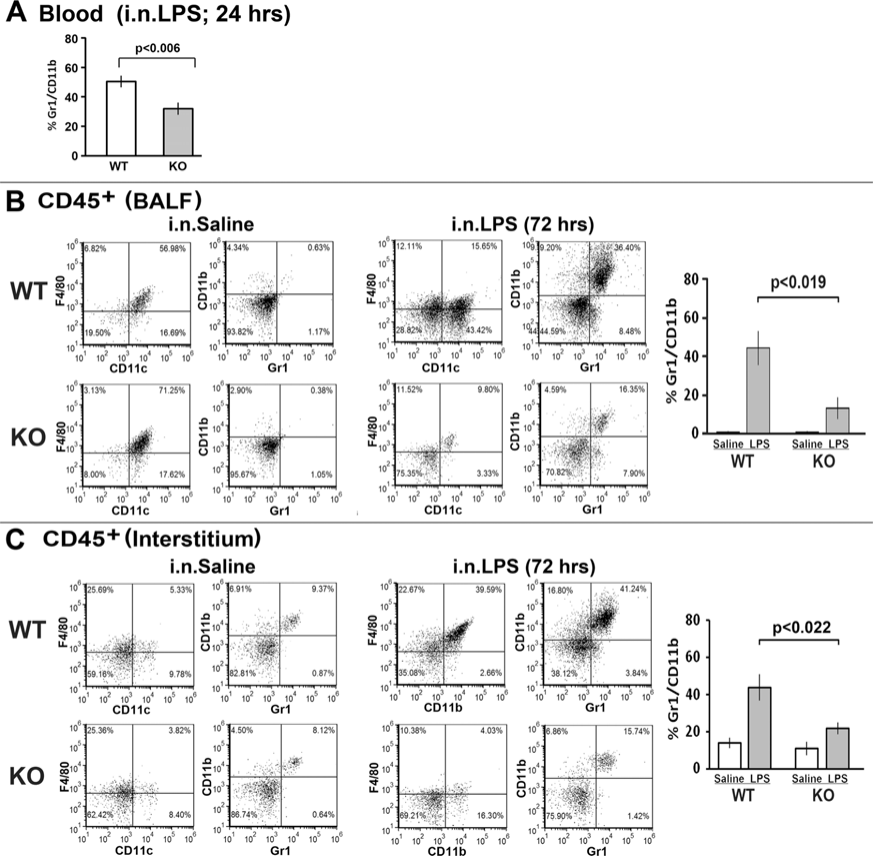
The α7 knock-out (α7^KO^) mice do not elicit a normal lung inflammatory response upon i.n. challenge with LPS. Mice (α7^WT^ or α7^KO^) were i.n. challenged with LPS (250 μg/mouse in 30 μl Saline) or saline (30 μl per mouse). For all experiments the bar graphs on the right represent quantification of the results from three independent experiments (12 animals (4–5 mice per experimental group) total per α7^WT^ or α7^KO^ genotypes, respectively) with error bars representing the standard error of the mean (SEM). Statistical significance was determined with the Student’s t-test. (A) 24 hours post-challenge small samples of blood (50 μl) were collected and assessed for CD11b^+^Gr1^+^ cells. In terms of circulating cells in the blood, the α7^KO^ exhibited an overall reduced number. To measure the lung, mice were sacrificed 3 days after LPS-challenge and BALF (B) and interstitial cells (C) were isolated and measured (see Fig. 2 and the Methods). The total number of cells isolated from the BALF of saline treated (i.n.) WT and α7^KO^ mice was similar (360,000 from the WT mice and 320,000 from the α7^KO^ mice). However, upon LPS challenge the number of cells isolated from WT BALF increases to an average of 740,000 cells/mouse in comparison to 450,000 cells from the BALF of the α7^KO^ mouse (n = 4 mice per group). These cells were stained for CD45, F4/80, CD11c, CD11b and Gr1 markers and analyzed by FACS. Flow cytometry data for isolated interstitial cells was gated on CD45^+^ cells. In the interstitium, similar to the BALF, fewer CD45^+^ cells were obtained from the α7^KO^ mouse (2.2 x 10^6^ cells from the WT versus 0.9 x 10^6^ cells from the α7^KO^). Virtually all cells in the BALF are CD45^+^.

We previously reported that in mice the response by skin to croton oil or ultraviolet radiation (UVB) was substantially greater in terms of inflammatory cytokine production by the α7^KO^ when compared to WT mice [12,13]. This was consistent with the absence of all α7 functions enhancing the inflammatory responses. However, little is known about lung inflammatory responses of the α7^KO^ mouse. One study reported that acid-induced lung injury in the α7^KO^ mouse resulted in a two-fold increase in lung edema and vascular permeability shortly after intra-tracheal acid administration [18]. Our goal was to measure the response to LPS administration (i.n.) in the α7^KO^ and then compare this to the α7 point mutation mouse line (α7^E260A:G^). The α7^KO^ mouse is bred through crossing α7^KO^ heterozygote mice and therefore, control mice (WT) for these experiments are age matched littermates as determined through genotyping. Both control α7^WT^ and α7^KO^ mice were administered i.n. LPS and twenty-four hours later the percentage of Gr1^+^CD11b^+^ was measured in a small sample of blood. There was an increase in Gr1^+^CD11b^+^ cells in the blood of both the α7^KO^ and α7^WT^ mice since naïve or saline treated mice had between 7% and 15% Gr1^+^CD11b^+^ cells in the blood (Fig. 3). However, unlike the hyper-responsiveness of the α7^KO^ skin to inflammatory challenge [12,13], the α7^KO^ mouse elicits a muted response to i.n. LPS (Fig. 3) as determined by reduced blood levels of Gr1^+^CD11b^+^ cells compared to similarly treated α7^WT^ mice (Fig. 3A). On day 3 post i.n. LPS challenge, α7^WT^ and α7^KO^ mice were sacrificed and the cellular composition of BALF and interstitial compartments was measured. Again, there were significantly fewer Gr1^+^CD11b^+^ cells in the BALF isolated at 72 hrs post-LPS from the α7^KO^ mice compared to α7^WT^ mice. Similarly, CD45^+^ interstitial cells from the α7^KO^ mice challenged with LPS had significantly fewer Gr1^+^CD11b^+^ cells compared to interstitium of α7^WT^ mice challenged with LPS (Fig. 3B, C). This observation was confirmed in at least 3 independent experiments. In each experiment, with 3–4 mice were included in each experiment group.

**Fig 4:**
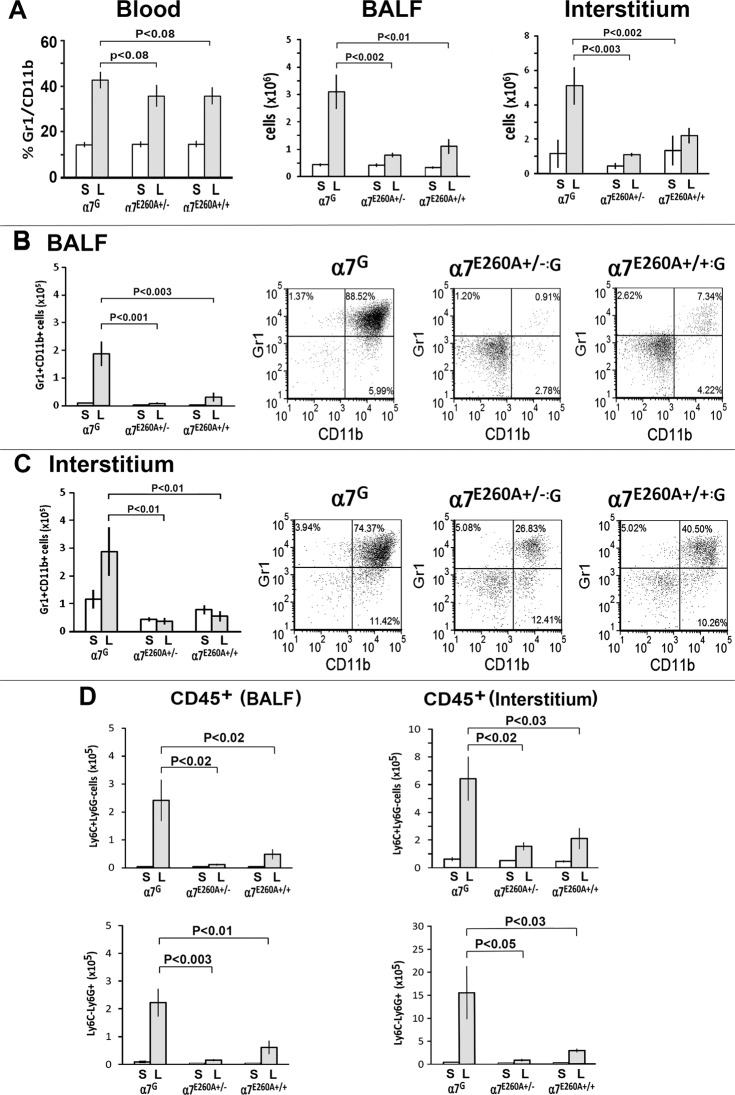
The response of α7^G^, α7^E260A:G^ heterozygote and α7^E260A:G^ homozygote mice to i.n. LPS challenge. Mice (α7^G^, heterozygote α7^E260A+/-:G^ or homozygote α7^E260A+/+:G^) were challenged with i.n. LPS (250 μg/mouse in 30 μl Saline) or saline (30 μl per mouse). For all experiments the results are shown as scatter plots and quantitated with bar graphs reflecting the results of three independent experiments (4 to 5 mice per experimental group). Error bars represent standard error of the mean (SEM) and statistical significance was determined using the Student’s t-test. (A) a small sample of blood (50 μl) was collected 24 hours LPS post-challenge and assessed for CD11b^+^Gr1^+^ cells (left panel). On day 3 after challenge, mice were sacrificed and BALF and interstitial cells isolated as described above and in the Methods for determination of total number of nucleated cells (A, middle and right panels). (B) BALF cells and (C) interstitial CD45^+^ cells isolated at 72 hrs post-challenge from α7^G^, α7^E260A+/-:G,^ α7^E260A+/+:G^. (D) BALF and interstitial CD45^+^ cells are shown and further identified by markers for monocyte/macrophages (Ly6C) and PMN (Ly6G).

We next determined the effect of i.n. LPS induced lung inflammatory response in the α7^E260A^ mouse. Each experiment described was performed at least 3 times and n = 4 to 5 mice individually measured per experiment for BALF and interstitial cells. The normal CD45^+^ cellular composition in the BALF (CD11c^+^) and lung interstitium (CD11b^+^) of naive α7^E260A:G^ homozygote and heterozygote mice is equivalent to naive α7^G^ (control) mice (data not shown). Mice (α7^G^ and α7^E260A:G^) were administered i.n. LPS and a small blood sample was obtained 24 hrs later to determine if a systemic response occurred as determined by the influx of inflammatory cells into the blood. In a non-LPS treated mouse, the percent of Gr1^+^CD11b^+^ cells in the blood is typically between 7% and 15% of the nucleated cells (Fig. 4A, left panel). At 24 hrs after i.n. LPS, blood levels of Gr1^+^CD11b^+^ cells in all mice increased to 30% or more with no statistically significant difference between mouse strains (α7^G^ to α7^E260A:G^). This result demonstrates that signaling after the i.n. LPS assault to initiate migration of inflammatory cells is normal in the α7^E260A:G^ mouse. However, lungs harvested at 72 hrs post-LPS administration had significantly fewer α7^E260A:G^ nucleated cells in both the BALF and interstitium when compared to the control α7^G^ (Fig. 4 A, right panels). Significantly fewer Gr1^+^CD11b^+^ cells migrated into the BALF and interstitium of LPS challenged α7^E260A:G^ mice compared with α7^G^ animals (Fig. 4B, C). Of the cells in the BALF and interstitium that are affected, Ly6C^+^Ly6G^-^ cells (monocyte/macrophages) and Ly6C^-^Ly6G^+^ (neutrophils) are similarly reduced in both the BALF and interstitium of the α7^E260A:G^ mice (Fig. 4D). Thus, the point mutation associated with α7 calcium permeability significantly reduces the inflammatory cell migration (macrophages and PMNs) from the blood to the lungs of mice exposed to LPS.

### Cell-specific effects on recruitment to the lung after i.n. LPS challenge are revealed in bone marrow chimeric mice

**Fig 5:**
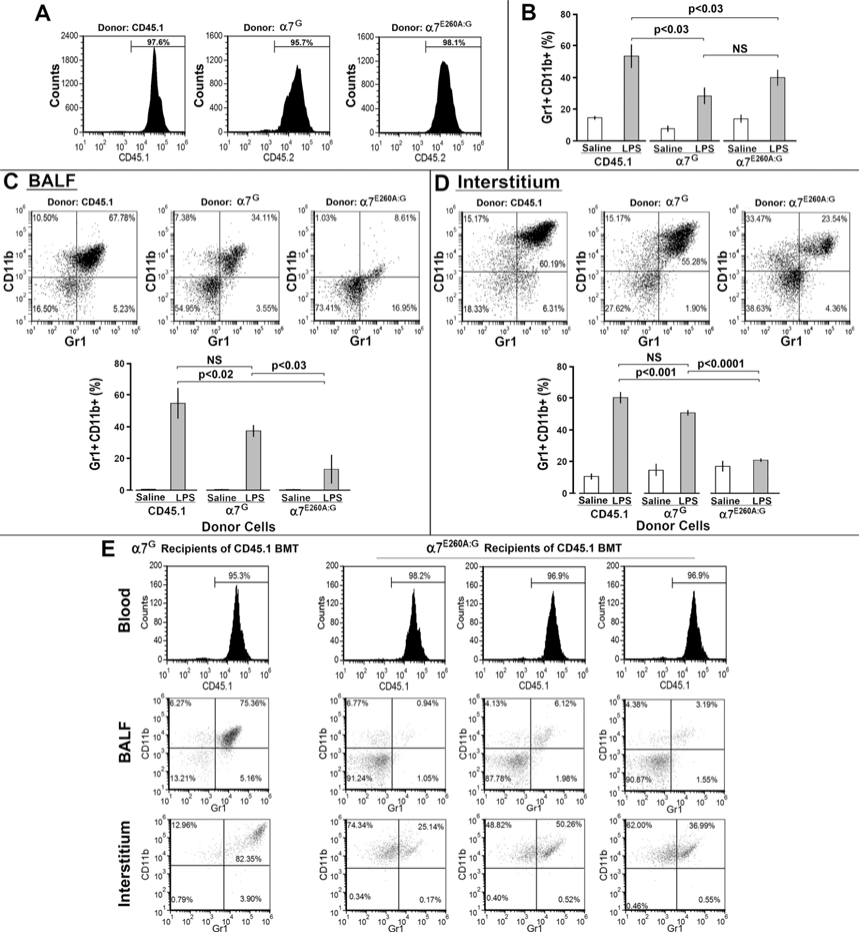
The response of chimeric α7^G^/α7^E260A:G^ mice to i.n. LPS. (A) Bone marrow cells were isolated from either B6.SJL (CD45.1 allele), α7^G^ (CD45.2 allele), or α7^E260A:G^ (CD45.2 allele) mice. B6.SJL (CD45.1 allele) mice were lethally irradiated with a dose of 2 x 6Gy, and 5 x 10^6^ cells per mouse were injected retro-orbitally into these recipients. Blood samples at 12 weeks post reconstitution show that greater than 95% of the nucleated cells of the blood were of donor origin. (B) Thirteen weeks post-BMT mice were challenged with either i.n. saline or i.n. LPS as above and a blood sample was obtained at 24 hrs post-challenge. All engrafted mice responded to LPS as measured by an increase in Gr1^+^CD11b^+^ cells in the blood. (C) BALF was obtained at 72 hrs post LPS challenge by lavage of individual mice and stained for Gr1^+^CD11b^+^. The total number of cells isolated from the BALF of the recipient mice repopulated with CD45.1 donor BM challenged with LPS was 2.4 x 10^6^ cells and 2.1 x 10^6^ cells from recipients repopulated with the α7^G^ donor BM, while the recipients of the α7^E260A:G^ BM had only 0.7 x 10^6^ BALF cells following LPS challenge. Similarly, the number of CD45^+^ cells recruited to the interstitium of the CD45.1 and α7^G^ mice was equivalent while the number of CD45.1 BM cells recruited to the interstitium of the α7^E260A:G^ mice was reduced. The bar graphs represent the average response of the mice to LPS and significance calculated using the Student’s t-test. (D) Following removal of BALF, interstitial tissue cells were isolated and stained for flow cytometry. Bar graphs reflect the average population response as indicated. (E) Bone marrow cells were isolated from B6.SJL (CD45.1 allele) mice and injected into lethally irradiated α7^G^ or α7^E260A+/+:G^ mice (CD45.2 allele). At 12 weeks recipient mice were fully reconstituted with CD45.1 cells (Blood, top panel). At week 13, recipient mice were challenged with either i.n. saline or LPS and sacrificed at 72 hrs for isolation of BALF or interstitium. A representative response in the BALF and interstitium (gated on CD45.1 cells) of the α7^G^ cellular response (CD45.1 donor cells transplanted into α7^G^ recipient mice) is shown in the left column. The total number of BALF cells from α7^G^ recipients of CD45.1 BM averaged 1.8 x 10^6^ cells/mouse following LPS challenge while the BALF of α7^E260A:G^ recipients of CD45.1 cells contained significantly less (0.5 x 10^6^ cells/mouse). CD45^+^ interstitial cell numbers also reflected this difference in total number of recruited cells with significantly fewer CD45.1 cells recruited to the lungs of the α7^E260A:G^ recipients (2.3 x 10^6^ versus 1.5 x 10^6^, respectively). The LPS response of the CD45.1 recipients of α7^E260A:G^ BM is shown for 3 individual mice (right panels).

The inability to recruit cells to the lung after i.n. LPS in the α7^E260A:G^ mouse was examined in reciprocal bone marrow (BM) chimeric mice. Lethally irradiated recipient B6.SJL mice expressing the CD45.1 allele were reconstituted with B6.SJL (CD45.1 allele) BM cells or CD45.2^+^ BM cells from either α7^G^ donors or α7^E260A:G^ donors, respectively. Recipients were periodically bled to confirm successful bone marrow engraftment. Results in Fig. 5A show that by week 12 post bone marrow transplant (BMT) the majority of cells in the blood of the B6.SJL/CD45.1 recipients reconstituted with B6.SJL/CD45.BM cells were CD45.1^+^ (as expected for the control), and B6.SJL/CD45.1 recipients reconstituted with CD45.2 BM cells were CD45.2^+^ indicating that mice receiving either CD45.1, α7^G^ or α7^E260A:G^ (homozygous) bone marrow were stably engrafted. No difference in the total number of blood cells was detected between these mouse groups.

Bone marrow reconstituted mice were challenged with either i.n. saline or i.n. LPS and recruitment of cells to the blood was measured at 24 hrs. Results demonstrate that i.n. LPS challenged recipient mice demonstrated an essentially equivalent increase in percentage of blood CD45.1^+^Gr1^+^CD11b^+^ or CD45.2^+^Gr1^+^CD11b^+^ cells (Fig. 5B). B6.SJL/CD45.1 allele mice engrafted with syngeneic CD45.1 donor cells did respond better to LPS with respect to increased Gr1+/CD11b+ recruitment to the blood than mice receiving the CD45.2 cells. At 72 hours post-i.n. LPS administration, lungs of BMT mice were harvested and the cellular composition of BALF and interstitium was measured as shown in Fig. 5C and D. Recipient mice engrafted with either B6.SJL/CD45.1 or α7^G^ BM demonstrated a response to LPS as measured by the presence of recruited Gr1^+^CD11b^+^ cells into BALF. While there is a response to the i.n. LPS by the α7^E260A:G^ mouse as observed by recruitment of these cells into BALF and interstitium, this response is significantly less than the response of the donor α7^G^ cells (Fig. 5C, D). Quantitation of these results shows that only BMT mice reconstituted with B6.SJL/CD45.1 or α7^G^ BM cells had significant influx of Gr1^+^CD11b^+^ cells to the BALF and interstitium post-i.n. LPS challenge. This supports the conclusion that the α7^E260A:G^ hematopoietic cells have a reduced capacity to respond to signals elicited in the lung upon inflammatory challenge.

To further explore the role of α7 signaling by lung CD45^-^ cells after challenge with i.n. LPS, we analyzed the response of chimeric mice generated from B6.SJL/CD45.1 BM cells (donor cells) transplanted into α7^G^ or α7^E260A:G^ irradiated recipients. As shown in Fig. 5E, the mice were fully reconstituted with donor BM as evidenced by the presence of cells (> 95%) expressing the CD45.1 allele in the blood. In response to i.n. LPS the α7^G^ mice reconstituted with CD45.1 BM demonstrate a significant increase in Gr1^+^CD11b^+^ cells in both the BALF and interstitium upon i.n. LPS administration (Fig. 5E). However, in α7^E260A:G^ recipients (3 individual responses shown), CD45.1 BM cells were poorly recruited to the BALF and a reduced number of cells are also measured in the interstitium. This result suggests that the α7^E260A:G^ interstitial cells (CD29^+^CD31^-^) exhibit reduced effectiveness in recruiting inflammatory cells to the lung following i.n. LPS administration. Therefore, the point mutation in the α7^E260A:G^ mouse contributes to both the hematopoietic and non-hematopoietic cell response of the lung to an inflammatory challenge.

### RNA expression by both CD45^-^ and CD45^+^ lung cells to i.n. LPS is altered in a7^E260A:G^ mice

**Fig 6:**
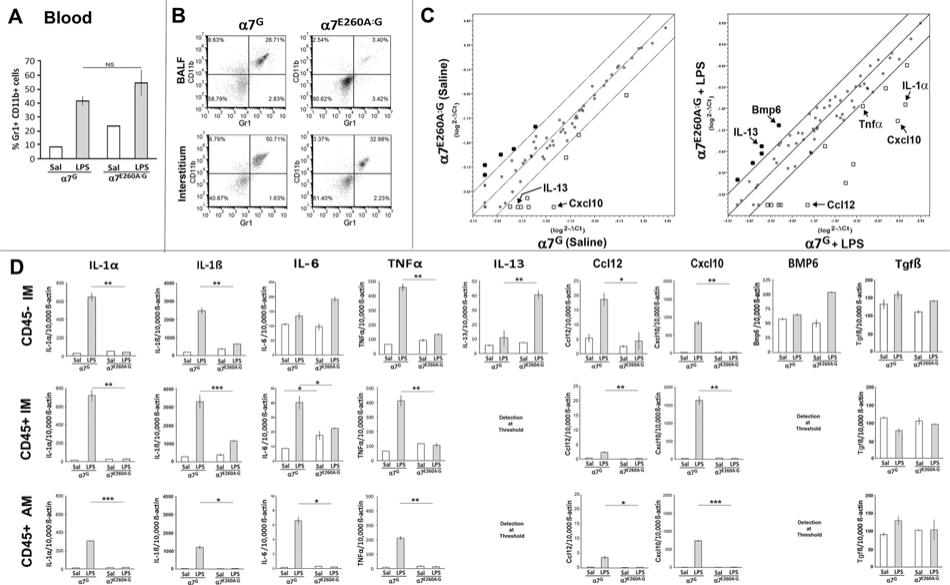
Gene expression analysis of lung cells from α7^G^ or α7^E260A+/+:G^ mice. Mice were i.n. challenged with either saline or LPS and harvested 18–20 hrs later. (A) Blood was collected at 18 hrs post-saline or LPS challenge to determine the number of Gr1^+^CD11b^+^ cells present. Error bars reflect +/- SEM. NS is non-significant as determined by the Student’s t-test. (B) BALF cells were isolated following lung lavage and interstitial cells were obtained from the digested lung tissue. A sample of these cells preparations were analyzed by flow cytometry for the presence of Gr1^+^CD11b^+^ cells. Note the substantial decrease in response in the α7^E260A:G^ double-positive cell populations compared to the control. (C) RNA was isolated from the remaining cells in these groups and tested for cytokine and chemokine transcripts using a SABioscience gene array kit (see Methods). The boundary lines shown indicate a difference of 4-fold in transcript number. Some points are identified are indicated. (D) In separately performed experiments the changes in RNA expression as indicated in (C) were confirmed using specific real-time PCR primer analysis (taqMan probes; see Methods). Results are presented as transcripts per 10,000 beta-actin transcripts. *p < 0.05, **p < 0.01, *** p < 0.001.

The analysis of gene expression kinetics revealed that 18 to 24 hrs was an optimal time to measure the transcriptional response in lung tissues of i.n. LPS challenged mice (not shown). The α7^G^ and α7^E260A:G^ mice were i.n. challenged with LPS and 18 hrs later a small amount of blood was taken to determine whether challenged mice had an influx of inflammatory Gr1^+^CD11b^+^ cells to the blood. Mice receiving 30 μl of saline (i.n.) were used as controls. Two hours later (20 hrs post LPS i.n.) mice were sacrificed and BALF, interstitial CD45^+^ and CD45^-^ cells were isolated. As expected, both the α7^G^ and the α7^E260A:G^ mice respond to i.n. LPS with an increase in Gr1^+^CD11b^+^ cells in the blood (Fig. 6A). The α7^G^ mice had significant numbers of Gr1^+^CD11b^+^ cells present in both BALF and interstitium, however, the influx of Gr1^+^CD11b^+^ cells into the α7^E260A:G^ interstitium and BALF was reduced substantially (Fig. 6B). A transcription profile of mouse chemokines and cytokines of CD45^+^ and the CD45^-^ cells of the interstitium (isolated with anti-CD45 microbeads) and CD45^+^ from the BALF was measured utilizing SABiosciences arrays (Methods). The purity of the cell preparations from which RNA was isolated was greater than 95%. The array measures a panel of chemokines and cytokines (Methods) and a typical result from multiple analyses is shown in Fig. 6C for the CD45^-^ fraction of the interstitial cells. Among the genes induced to at least a 4-fold a lesser extent compared to the α7^G^ mouse were Cxcl10, IL-1α, Ccl12 and TNFα. In the CD45^-^ fraction, IL-13 and BMP6 were selectively increased.

To confirm results in separate experiments, the RNA levels were measured by applying selected real-time PCR using Taqman gene probes (Fig. 6D). Similar to the array results, IL1α, TNFα, Ccl12 and Cxcl10 genes were induced to significant levels in CD45^-^ interstitial cells from the α7^G^ (WT) mouse challenged with LPS but not in CD45^-^ interstitial cells from the LPS stimulated α7^E260A:G^ mice. Additionally, we determined that CD45^-^ cells from LPS challenged α7^G^ mice expressed significantly higher levels of IL-1β gene expression while IL-6 was induced more in the α7^E260A:G^ mouse, TGFβ transcripts were approximately equivalent in both strains, and BMP6 gene expression levels were slightly elevated in the α7^E260A:G^ mouse CD45^-^ interstitial cells. We also determined transcript levels in the CD45^+^ interstitial and BALF cells as shown in Fig. 6D. Similar to the CD45^-^ cell transcripts we observe the same trends, however, differences in the magnitude of response is in accord with the cell type. For example, CD45^+^ cells make more IL-1α and TNFα while CD45^-^ cells make more BMP6. Notable differences between CD45^-^ and CD45^+^ transcripts are IL-6 and IL-13. For IL-6, we measured more transcripts, but poor induction, in the CD45^-^ cells than in the CD45^+^ cells. Further, the IL-6 response of the CD45^-^ cells from the α7^E260A:G^ response was greater than the α7^G^ CD45^-^ response. Further, when IL-13 gene expression was analyzed in CD45^-^ interstitial cells isolated from LPS challenged α7^G^ and α7^E260A:G^ mice, we found that CD45^-^ cells from α7^E260A:G^ mice upregulated IL-13 gene expression in response to LPS challenge (Fig. 6D). We did not observe significant IL-13 gene expression increases in the α7^G^ CD45^-^ cells nor the in any CD45^+^ cells. The recruitment of T-cells, a major CD45^+^ cell in the lung capable of producing IL-13, does not occur to a significant level in this model of LPS administration which accounts for the lack of IL-13 transcripts in this cell population (Gr1^+^CD11b^+^).

## Discussion

In this study, we have described the impact of a point mutation in the nicotinic acetylcholine receptor α7 on the inflammatory response by the lung to intranasal LPS. Hematopoietic cells in mice expressing WT α7^G^ or the α7^E260A:G^ point mutation both respond to i.n. LPS challenge as demonstrated by equivalent recruitment of hematopoietic inflammatory cells into the blood. However, thereafter trafficking of the α7^E260A:G^ inflammatory cells into both the lung interstitial and alveolar compartments is significantly reduced, both in total cell number and the percentage of inflammatory cells such as PMNs (Ly6G^+^), macrophages (CD11b^+^) and granulocytes (Gr1^+^). This decrease appears to be due to both the inability of CD45^+^α7^E260A:G^ cells to be recruited to the inflammatory site and the failure of normal signaling by CD45^-^ population of the lung interstitial cells, some of which are α7^lin+^ (discussed below). Transcriptional differences between the α7^G^ and α7^E260A:G^ responses to i.n. LPS challenge include the reduced transcription of both chemokines and cytokines by CD45^+^ and CD45^-^ lung cells, but increased IL-13 and BMP6 expression by CD45^-^ cells of the α7^E260A:G^ mouse. Taken together, these findings reinforce the concept that convergence of tissue and cell-specific α7 functional pleiotropy is a significant component of how this receptor systemically modulates the diverse cellular responses to different environmental challenges [27].

In previous findings we reported [12,13] that in the α7^KO^ recruitment of hematopoietic cells to a local site of skin inflammation induced by either UV irradiation or croton oil was increased. Therefore, the finding reported here that the overall response of the α7^KO^ lung challenged with i.n. LPS exhibited a reduced inflammatory cell recruitment and response compared to the WT lung suggests that the modulation of inflammation by α7 exhibits a strong tissue specific response. This likely reflects the local cell-type specific expression and individual response of those cells to the inflammogen. In another model of acute lung injury, Su et al. [18] reported that acid-induced lung swelling of the lung is elevated in the α7^KO^ mouse. Further, these investigators demonstrated that mortality in the α7^KO^ is greater upon challenge with live *E*. *coli* following instillation into the lung. Inflammatory cell infiltration into the α7^KO^ lung in that study was not reported and therefore we cannot compare our results with the acid induced response. Our results suggest that α7 contributes to the inflammatory response through modulating a two-step process. The first step is revealed in the α7^KO^ where there is reduced recruitment of hematopoietic cells into the blood after inflammatory challenge, and the second step is seen in the α7^E260A:G^ mouse where recruitment to the blood is near normal but the subsequent trafficking of inflammatory cells into the target tissue (lungs) is compromised. Thus, the cellular activation and signaling induced through the α7 is required to mobilize cells from the blood to the lungs but not required for mobilization from the bone marrow. Further, the results of comparing α7^WT^ and α7^E260A:G^ signaling show that control of cell entry into the lung reflects the local effects produced by both hematopoietic and non-hematopoietic cells. This is supported by the results of bone marrow transfer experiments demonstrating that the hematopoietic derived cells of the α7^E260A^ mouse do not traffic as well as α7^WT^ cells, especially with regard to recruitment into BALF. Conversely, in mice harboring CD45^-^α7^E260A:G^ lung cells, the recruitment of α7^G^ hematopoietic cells is significantly reduced. Consequently, the non-hematopoietic cell response in the lung that is controlled by α7 is an important contributor to this inflammatory response. These findings are consistent with other reports showing that lung epithelial cells express α7 [6,16,17] and this extends those findings to include the impact by α7 on tissue-specific signaling by multiple cell types in addition to those of hematopoietic origin during orchestration of the full systemic response.

We examined the RNA profile of the α7^E260A:G^ mouse where the functional defect has been associated with the loss of calcium-mediated signaling systems while retaining expression and other functions [5,31–34]. While we cannot rule out the possibility that this mutation alters other functions of alpha7, additional issues such as those pertaining to non-specific compensation that are often more commonly associated with the complete absence of the gene product can be avoided. Our results confirm that α7 in the non-hematopoietic cell fraction (CD45^-^) are indeed important to the coordinated expression of cytokines and chemokines, of which several monocyte activators and chemotactic modulators are altered in the α7^E260A:G^ lung (e.g., IL-1α, IL-1β, TNFα, IL-13, Cxcl10, Ccl12 and others). The α7^E260A:G^ mouse also exhibits an elevated production of IL-13 by CD45^-^ cells of the lung interstitium. Since the LPS model of lung inflammation does not promote the recruitment of T cells (a major source of IL-13 when this occurs), the production of this cytokine by epithelium could have local effects on both macrophages and epithelial cells. For example, IL-13 can induce alternatively activated M2-like macrophages, often associated with type-2 immunity to Helminthes as well as allergic airway responses [37]. IL-13 has also been reported to be responsible for mucus production and differentiation of goblet cells. Mucus formation is exacerbated in cell culture systems by nicotine acting through α7 and additional nAChR subtypes [38]. Further, IL-13 could interfere with hyaluroninan binding to CD44 possibly interfering with adherence of trafficking monocytes and lymphocytes from the vasculature into the lung [39–41]. Thus, the long term effects of the α7^E260A^ mutation on lung pathology could be highly specific to this particular role in the normal local inflammatory response leaving its impact on the consequences of other immune mediated challenges, such as in asthma where IL-13 is an important mediator of the response. Changes in RNA expression by the CD45^+^ cells isolated from LPS challenged lungs of the α7^WT^ and α7^E260A:G^ mice most likely represents changes in the cellular infiltration into the challenged lungs. As such, future studies will require that specific cell types be isolated from the lung (e.g., CD11b^+^, Gr1^+^) prior to RNA profiling to determine the impact of the α7^E260A^ mutation in immune cell function. The RNA data presented for the CD45^+^ cells suggests only that the inflammatory milieu in the α7^E260A^ mice is not equivalent to the α7^WT^ response.

How the α7^lin+/-^ lineage ratio is determined and how varied ratios quantitatively impact on the systemic inflammatory response remains to be answered. As we reported previously, upon measuring the percent of cells in the blood that are of the α7 lineage, the range we have observed is between 5% and 50%. One possibility is that this ratio reflects the origin of two separate but convergent cell lineages that produce phenotypically and functionally similar cells within the tissue structure. The expression of α7 begins at E9.0 in rhombomeres 3 and 5 and it expands rapidly thereafter to include a broad spectrum of cells in tissues throughout the body that exhibit α7-lineage. Thus it is possible that early α7 expression identifies one of the subsets of cells that populate the hematopoietic system. This is reminiscent of the fate of Pax7 and Pax3 cells that while of neural crest origin contribute to cells in tissues including the fat, skin epithelium, muscle and lung bronchioles among many others [42]. Thus the differential contribution by non-traditional cell lineages to the individual cells of a tissue and consequently in this case to the response to an inflammogen would in part be calibrated by the ratio of α7-lineage composition. Regardless of their origin, these findings raise several possibilities regarding how early defects in cell migration or survival could impact upon the cell composition and the regulation of inflammatory processes. The intriguing observation that a minority population of cells (α7^lin+^) appears to selectively be enriched in the lung following i.n. LPS and impacts the response of the majority of the cells has significant implications in understanding the modulatory effect of this receptor. Future studies should reveal how the individualized α7-lineage cell ratio contributes to the pattern and magnitude of organ specific inflammation.

Ongoing experiments in which these mice are exposed to cigarette smoke (CS) and nicotine will directly answer many questions, and provide insights into the molecular mechanisms modulated by α7 that are more diverse and potentially highly tissue specific. The impact of long term cigarette smoke in the α7^E260A^ mouse once determined will aid in our understanding of the tissue specific mechanisms through which nicotine promotes or inhibits inflammatory processes.
